# Selective carboxylation of reactive benzylic C–H bonds by a hypervalent iodine(III)/inorganic bromide oxidation system

**DOI:** 10.3762/bjoc.14.94

**Published:** 2018-05-16

**Authors:** Toshifumi Dohi, Shohei Ueda, Kosuke Iwasaki, Yusuke Tsunoda, Koji Morimoto, Yasuyuki Kita

**Affiliations:** 1 College of Pharmaceutical Sciences, Ritsumeikan University, 1-1-1 Nojihigashi, Kusatsu, Shiga 525-8577, Japan. Tel: +81-77-561-4908,; 2 Research Organization of Science and Technology, Ritsumeikan University, 1-1-1 Nojihigashi, Kusatsu, Shiga 525-8577, Japan. Tel & Fax: +81-77-561-5829

**Keywords:** carboxylic acids, C–H activation, iodine, oxygenation, radicals

## Abstract

An oxidation system comprising phenyliodine(III) diacetate (PIDA) and iodosobenzene with inorganic bromide, i.e., sodium bromide, in an organic solvent led to the direct introduction of carboxylic acids into benzylic C–H bonds under mild conditions. The unique radical species, generated by the homolytic cleavage of the labile I(III)–Br bond of the in situ-formed bromo-λ^3^-iodane, initiated benzylic carboxylation with a high degree of selectivity for the secondary benzylic position.

## Introduction

The oxidative activation of a C(sp^3^)–H bond in organic molecules to directly install various functional groups and new carbon–carbon networks is a topic of interest for researchers engaged in modern synthetic chemistry [1–8]. Benzylic oxidation is of particular interest because it is a convenient direct approach to arylcarbonyl compounds; it has a long history of research and development, and thus is included among the well-investigated C(sp^3^)–H transformations [9–12]. To widen the scope, recent studies and reaction systems have been further elaborated to include elegant C–H coupling methodologies. Several important researches that provide a new benzylic C–H coupling strategy have been reported over the past few years, involving the promising catalytic activities of metal complexes [13–14]. On the other hand, reports aimed at realizing efficient and selective metal-free C(sp^3^)–H transformations are rather limited; however, investigations by several research groups are still ongoing [15–30].

**Scheme 1:**
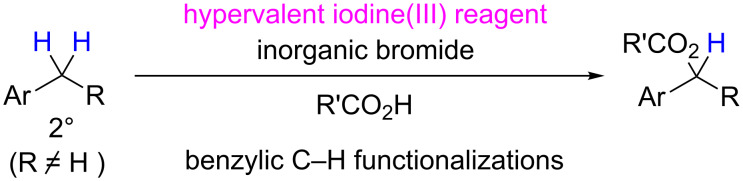
Hypervalent iodine(III)-induced benzylic C–H functionalization for oxidative coupling with carboxylic acids.

Hypervalent iodine reagents are now widely accepted as a safe replacement for certain heavy-metal oxidizers, such as lead, mercury, and thallium-based salts, due to their low toxicities, high stabilities, operational simplicities, and many other user-friendly characteristics [31–32]. By virtue of their wide array of reactivity patterns, the controllable radical and single-electron-transfer (SET) reactivities [33–37] allow selective activation of the benzylic C(sp^3^)–H bond for oxidative functionalization and coupling reactions. Initially, the SET oxidation ability of pentavalent iodine reagents, especially *o*-iodoxybenzoic acid (IBX), in benzylic oxidations was recognized for displaying the new reactivities of hypervalent iodine reagents toward C(sp^3^)–H bonds [38–39]. By exploiting the radical behavior of trivalent iodine reagents discovered previously [40–41], the activation of trivalent iodine reagents, e.g., phenyliodine(III) diacetate (PIDA), phenyliodine(III) bis(trifluoroacetate) (PIFA), and iodosobenzene, has since become a popular choice for benzylic oxidations, which further expanded the scope and availability of methods for direct C–H functionalization and several coupling reactions [42–50]. As such, we reported aqueous benzylic oxidations using polymeric iodosobenzene in the presence of inorganic bromide and montmorillonite-K10 [51]. In addition, a radical C–H activation strategy, using nonaqueous hypervalent iodine(III)/inorganic bromide systems that can work in organic solvents, was developed for the novel synthesis of lactones via the intramolecular oxidative cyclization of aryl carboxylic acids at the benzyl carbon under transition-metal-free conditions [52]. Based on our previous research and general interest in the unique reactivity of hypervalent iodine(III)–Br bonds [53–56], we report the results of our extensive study and optimization of our radical C–H activation strategy for the intermolecular oxidative coupling between the benzylic secondary C–H bond and the O–H group of carboxylic acids (Scheme 1).

## Results and Discussion

Benzylic C–H carboxylation can provide a convenient route to benzyl esters from non-functionalized aromatic hydrocarbons, and thus has attracted continuous interest in the community of synthetic chemists. Significant advances for realizing such transformations have been made over the last decade, however, strategies that do not utilize a directing group to facilitate the activation of the benzylic C(sp^3^)–H bond are rare [21,57–62]. Furthermore, only a limited number of transition-metal-free methods have been reported; successful examples include the Wohl–Ziegler-type conditions [21], the sodium bromate system [57] for the conversion of benzylmethyl groups, and the use of 2,3-dichloro-5,6-dicyano-1,4-benzoquinone (DDQ) [58] or catalytic tetrabutylammonium iodide with *tert*-butyl hydrogen peroxide for reactions with a large excess of aromatic hydrocarbons [59]. Other than these excellent examples of metal-free methods, two protocols using a hypervalent iodine reagent were reported, both of which include the formation of benzyl radicals during the key initial reaction step. Togo and co-workers developed a reaction system consisting of stoichiometric amounts of PIDA with catalytic amounts of molecular iodine and *p*-toluenesulfonamide for the benzylic acetoxylation and benzoyloxylation of alkylbenzenes, where an in situ-generated sulfonamidyl radical is the essential radical mediator that effectively abstracts the benzylic hydrogen [49]. More recently, Maruoka et al. succeeded in the photolytic benzylic C–H bond oxygenation of alkylbenzenes initiated by the decomposition of PIFA to form the trifluoroacetoxy radical under visible light irradiation [50].

**Scheme 2:**
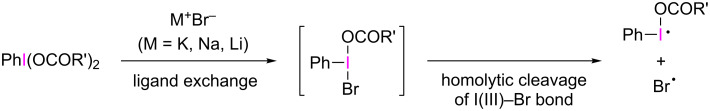
Radical reactivities of the I(III)–Br bond generated from PIDA.

Our approach for the generation of radical species for the benzylic carboxylation using a hypervalent iodine reagent relies on the unique reactivity of the hypervalent iodine(III)–bromine bond with the following mechanistic principles: As illustrated in Scheme 2, ligand exchange at the iodine(III) center of the phenyliodine(III) dicarboxylate with the bromide ion can gradually produce the corresponding bromo-λ^3^-iodane in the first step [63–64]. This unstable hypervalent iodine(III) species subsequently decomposes by facile homolytic cleavage of the I(III)–Br bond, generating the iodanyl and bromo radicals [51–52]. It appears that these radicals can then selectively abstract the benzylic hydrogen atom of organic substrates, even in the presence of a wide variety of functional groups, such as electrophilic aromatic rings, non-acidic carbonyl groups, and suitable oxygen, nitrogen, and sulfur functionalities. Carbonyloxy radicals derived from typical hypervalent iodine(III) carboxylates by photolysis and other conditions [65–68] are known to undergo irreversible decarboxylation [69–70]. Therefore, the formation of carbonyloxy radicals must be avoided to prevent non-productive side reactions and achieve the desired benzylic C–H transformations for an extended series of carboxylic acids.

Based on these considerations, we performed an optimization study using 1-ethyl-4-methoxybenzene (**1a**) as a model substrate for the oxidative C–H coupling of the benzyl group with acetic acid derived from PIDA (Table 1). Reactions using PIDA with finely powdered inorganic bromide in degassed dichloromethane containing 0.1 M of the substrate were first examined at room temperature. The use of potassium bromide [52] afforded modest yields of the carboxylation product **2a** (Table 1, entry 1). Interestingly, a dramatic influence was observed when altering the bromide source to other types; the use of lithium bromide or organic bromides, e.g., bromotrimethylsilane and tetraethylammonium bromide, instead of the potassium salt, were unsuccessful in forming the carboxylate **2a** (Table 1, entries 2–4). The reason for this behavior was thought to be because lithium bromide or organic bromides in combination with PIDA generated the electrophilic ‘Br^+^’ species [71] and molecular bromine [72], or hypobromite and bisacetoxy bromate(I) [73], respectively, rather than the desired bromo radical. As a result, bromination at the aromatic ring of substrate **1a** occurred when LiBr was used (Table 1, entry 2), while no reaction was observed in the other two trials using organic bromides (Table 1, entries 3 and 4). At the boiling temperature of dichloromethane, ca. 40 °C, the reaction time was shorter (Table 1, entry 5). Aiming to improve the yield of the reaction, we then examined other inorganic bromides, among which sodium bromide was the most promising, and product **2a** was obtained in 76% yield (Table 1, entry 6). By adding extra acetic acid, the benzylic acetoxylation was further improved to provide an 86% yield of product **2a**. Since most of the sodium bromide was present as a precipitate in the flask, the reaction was found to work even with catalytic amounts of the bromide activator (Table 1, entry 8). Other reaction factors, such as solvent, concentration, reaction time, and reagent quantities, were screened and, eventually, the reaction conditions of entries 6 and 7, which consisted of 1.2 equiv of PIDA with 2 equiv of sodium bromide in dichloromethane (0.1 M of the reaction substrate) at 40 °C, were determined to be the best in terms of product yield. No reaction was observed in the absence of sodium bromide (Table 1, entry 9) and other representative hypervalent iodine(III) reagents, such as PIFA and PhI(OH)OTs, and pentavalent Dess–Martin periodinane and IBX, were inferior for this carboxylation when compared to PIDA.

**Table 1:** Reaction optimization of benzylic C–H acetoxylation using PIDA.

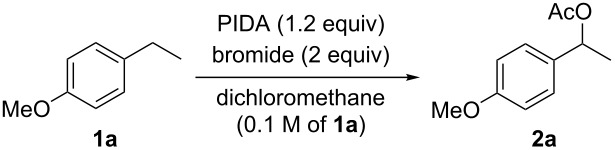			

entry	bromide	conditions	yield of **2a** (%)

1	potassium bromide	rt, 6 h	55
2	lithium bromide	rt, 20 h	11^a^
3	bromotrimethylsilane	rt, 20 h	nd
4	tetraethylammonium bromide	rt, 20 h	nd
5	potassium bromide	40 °C, 4 h	57
6	sodium bromide	40 °C, 4 h	76
7^b^	sodium bromide	40 °C, 3 h	86^c^
8^b^	sodium bromide^d^	40 °C, 3 h	72
9	none	40 °C, 3 h	nd

^a^Mainly bromination of the aromatic ring was observed. ^b^Acetic acid (20 equiv) was added. ^c^The reaction was performed on a 10 mmol scale. ^d^Catalytic amounts of sodium bromide were added (0.5 equiv). nd: not determined.

We then examined the reactivity of different benzyl groups under the optimized reaction conditions for the radical activation system for C–H acetoxylations (Table 2). When the reactions were performed using ethylbenzene (**1b**) and its derivatives without an electron-donating group (**1c**–**e**), the corresponding benzyl bromides were mainly obtained along with a small amount of the target C–H acetoxylation product; this byproduct formation might imply the intermediacy of these organic bromides before the production of benzyl acetates. Hence, the reaction system was modified to include zinc(II) acetate [74] for substrates **1b**–**d** and benzyl acetates **2b**–**d** were obtained in moderate to excellent yields after prolonged reaction times (Table 2, entries 1–4). Furthermore, iterative oxidative coupling at the aromatic and benzylic C–H position using hypervalent iodine chemistry is possible, and 1-ethyl-4-methoxy-3-thiocyanatobenzene (**1f**), prepared from 1-ethyl-4-methoxybenzene (**1a**) through the hypervalent iodine(III)-induced aromatic cation radical coupling with thiocyanate [75–76], similarly acetoxylated under the standard reaction conditions without zinc(II) acetate (Table 2, entry 5). The acetoxylation at the activated benzyl carbon adjacent to an oxygen atom proceeded smoothly as shown by the formation of the *pseudo* acetal **2g** (Table 2, entry 6). Diphenylmethane and its derivatives were very good substrates for our system and were less sensitive to the electronic effects of the aromatic ring substituents than in other previously described compounds, showing higher reactivity and chemoselectivity of the benzylic position (see ref. [51]). The reactions of substrates **1h**–**j** proceeded without the use of zinc(II) acetate (see Table 2, entries 7–9 versus entry 2).

**Table 2:** Substrate screening for benzylic C–H acetoxylation by the PIDA/NaBr system.^a^

entry	substrate	product	time	yield of **2** (%)

	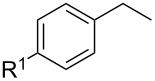 **1b–e**	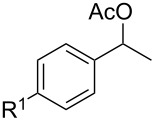 **2b–e**		
		
1	**1b** (R^1^ = H)	**2b** (R^1^ = H)	20 h	55^b^
2	**1c** (R^1^ = Ph)	**2c** (R^1^ = Ph)	20 h	91^b^
3	**1d** (R^1^ = Br)	**2d** (R^1^ = Br)	20 h	65^b^
4	**1e** (R^1^ = CO_2_CH_3_)	**2e** (R^1^ = CO_2_CH_3_)	48 h	nd^c^

5	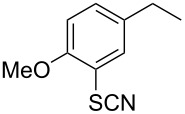 **1f**	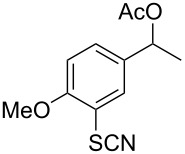 **2f**	20 h	62
6	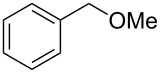 **1g**	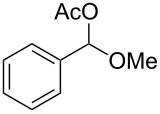 **2g**	4 h	68

	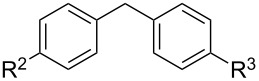 **1h**–**j**	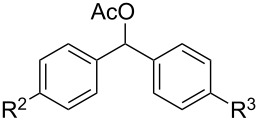 **2h**–**j**		
		
7	**1h** (R^2^ = R^3^ = H)	**2h** (R^2^ = R^3^ = H)	4 h	90
8	**1i** (R^2^ = R^3^ = F)	**2i** (R^2^ = R^3^ = F)	4 h	93
9	**1j** (R^2^ = Ph, R^3^ = H)	**2j** (R^2^ = Ph, R^3^ = H)	4 h	45

10	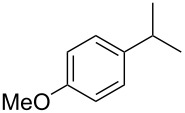 **1k**	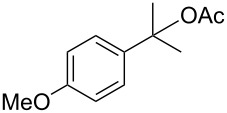 **2k**	4 h	nd

^a^Conditions: PIDA (1.2 equiv), sodium bromide (2 equiv), and acetic acid (20 equiv) in dichloromethane (0.1 M of substrate **1**) at 40 °C. nd: not determined. ^b^Zinc acetate (1 equiv) was added for the conversion of the benzyl bromide byproduct to the desired benzyl acetates **2b**–**d**. ^c^The corresponding benzyl bromide was obtained in 60% yield.

**Scheme 3:**
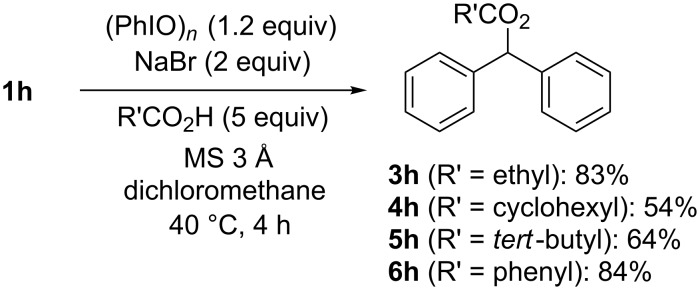
Benzylic C–H carboxylations by the iodosobenzene/NaBr system.

The installation of other carboxylic acids, such as propionic acid, cyclohexyl carboxylic acid, pivalic acid, and benzoic acid, were also possible by simply replacing PIDA with iodosobenzene (Scheme 3). Here, the addition of 3 Å molecular sieves was essential for removing water derived from the iodosobenzene and to suppress the benzylic oxidation forming aryl ketones [52]. Note that the successful coupling of a range of secondary and tertiary carboxylic acids now supports the direct and selective C–H bond activation at the benzyl position by avoiding the formation of carbonyloxy radicals, which are susceptible to decarboxylation. In addition, it was revealed that more acidic benzoic acids were also suitable substrates for our method. However, even more acidic acids, such as trifluoroacetic acid and methanesulfonic acid, were not effectively introduced by our benzylic C–H carboxylation procedures.

**Scheme 4:**
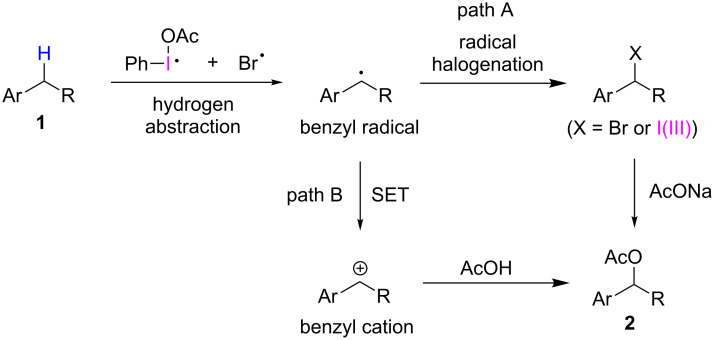
Outline of the proposed reaction mechanism for the PIDA/NaBr system.

We believe that the reaction mechanism involves a benzyl radical formation as the initiating step (Scheme 4). Either the iodanyl radical or the bromo radical may cause the H-atom abstraction generating the benzyl radical, and the resulting benzyl radical is trapped by the persistent bromo radical*,* giving rise to the observed benzyl bromide intermediate (path A). Alternatively, the benzyl radical formed in situ couples with the iodanyl radical to give the *pseudo* halide, benzyl-λ^3^-iodane, which is more reactive to nucleophilic substitution by a carboxylic acid [77–78]. Another possible course of the reaction mechanism assumes the formation of a benzyl cation, resulting from the SET oxidation of the benzyl radical, probably by the iodanyl radicals (path B).

**Scheme 5:**
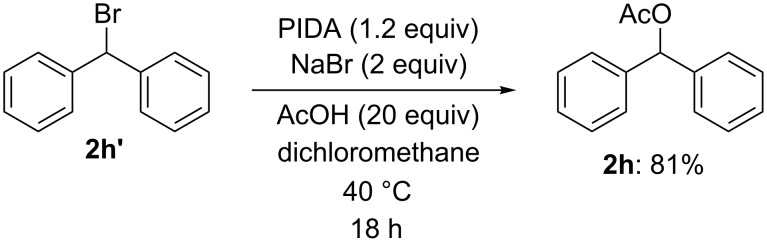
Reaction of benzyl bromide **2h’** under radical C–H acetoxylation conditions.

A significant point of interest in this study is understanding the relative reactivity of the iodanyl radical versus the bromo radical. To gain more information about this, we confirmed the chemoselectivity of our reaction system towards different classes of benzyl carbon atoms. To this end, the clear direction to the secondary benzylic position in substrates **1a–j** [51], and the lack of benzylic acetoxylation of the tertiary carbon in substrate **1k** (see Table 2) are noteworthy. As bromo radicals are known to readily abstract hydrogen atoms at the tertiary carbon centers [79], the results suggest a predominant participation of the iodanyl radical as the hydrogen atom abstractor for the selective secondary benzylic C–H bond activation in our reaction. It seems that the iodanyl radical is more reactive than the bromo radical and a large-size effect of the hypervalent iodine species establishes the selectivity over the electronically favored tertiary radical formation. A similar trend in the secondary-preferential hydrogen abstraction at the sp^3^ carbons was recently reported by Maruoka and co-workers for the C–H oxidations of unreactive alkanes by iodanyl radicals [80]. Based on these observations, the reaction mechanism via path A that involves the formation of benzyl bromides (X = Br) seems to be more reasonable for our benzylic C–H carboxylation system based on the hypervalent iodine(III) reagent/inorganic bromide combination. This mechanistic course was also partially supported by the control experiment, whereby one of the separately prepared bromides, **2h’**, was gradually transformed into the corresponding acetate **2h** in good yield under the radical C–H acetoxylation reaction conditions (Scheme 5).

## Conclusion

In conclusion, we have described the optimization and scope of an oxidation system for benzyl C–H carboxylation that utilizes the radical reactivity of a hypervalent iodine(III) reagent produced under suitable conditions. The mechanistic information obtained in this study indicates that iodanyl radicals, generated by the homolytic cleavage of the labile I(III)–Br bond of in situ formed bromo-λ^3^-iodane, are the key initiators for this benzylic carboxylation, and they show a high degree of selectivity towards the secondary benzylic position under mild reaction conditions.

## Experimental

### Representative experimental procedure for the benzylic C–H acetoxylation by the PIDA/NaBr system

In a flame-dried two-necked round-bottomed flask, under nitrogen, phenyliodine(III) diacetate (PIDA, 193 mg, 0.6 mmol) and finely powdered sodium bromide (103 mg, 1.0 mmol) were subsequently added to a stirred solution of arylalkane **1** (0.50 mmol) and acetic acid (0.57 mL, ca. 10 mmol) in dry dichloromethane (5 mL). Then the mixture was vigorously stirred at 40 °C with or without zinc acetate (92 mg, 0.5 mmol). After checking the reaction completion by TLC, saturated aqueous sodium carbonate was added to the mixture and the resulting solution was stirred for an additional 5 min. The organic layer was separated, washed again with saturated aqueous sodium carbonate, then with dilute aqueous sodium thiosulfate, and was dried over anhydrous sodium sulfate. After removal of the solvents, the residue was subjected to column chromatography on silica gel (eluents: *n*-hexane/ethyl acetate) to give the benzyl acetate **2** in the indicated yield; the physical and spectral data of the reported compounds (**2a**–**d**, **g**–**j**) matched those of the authentic samples (see Supporting Information File 1).

**1-(4-Methoxy-3-thiocyanatophenyl)ethyl acetate (2f).** Yield 62% (156 mg). Obtained as a colorless oil; ^1^H NMR (CDCl_3_, 400 MHz) δ 1.51 (d, *J* = 6.8 Hz, 3H), 2.05 (s, 3H), 3.89 (s, 3H), 5.80 (q, *J* = 6.8 Hz, 1H), 6.88 (d, *J* = 8.3 Hz, 1H), 7.32 (dd, *J* = 8.3, 1.9 Hz, 1H), 7.53 (d, *J* = 2.0 Hz, 1H) ppm; ^13^C NMR (100 MHz, CDCl_3_) δ 21.3, 22.1, 56.3, 71.3, 110.3, 111.2, 113.3, 127.6, 128.7, 135.8, 156.0, 170.2 ppm; IR (KBr): 2984, 2158, 1741, 1603, 1499, 1370, 1291, 1242, 1208, 1062, 1023 cm^−1^; HRMS (MALDI): [M + Na]^+^ calcd for C_12_H_13_NO_3_SNa, 274.0507; found, 274.0508.

### Representative experimental procedure for the benzylic C–H carboxylation by the iodosobenzene/NaBr system

In a flame-dried two-necked round-bottomed flask, under nitrogen, iodosobenzene (132 mg, 0.6 mmol) and finely powdered sodium bromide (103 mg, 1.0 mmol) were subsequently added to a stirred solution of diphenylmethane (**1h**, 84 mg, 0.50 mmol) and the carboxylic acid (ca. 2.5 mmol) with freshly-dried molecular sieves 3 Å (ca. 300 mg) in dry dichloromethane (5 mL). Then the mixture was vigorously stirred at 40 °C. After checking the reaction completion by TLC, saturated aqueous sodium carbonate was added to the mixture and the resulting solution was stirred for an additional 5 min. The organic layer was separated, washed again with saturated aqueous sodium carbonate, then with dilute aqueous sodium thiosulfate, and was dried over anhydrous sodium sulfate. After removal of the solvents, the residue was subjected to column chromatography on silica gel (eluents: *n*-hexane/ethyl acetate) to give the benzylic C–H carboxylation product in the indicated yield; the physical and spectral data of the carboxylation products (**3h, 4h, 5h, 6h**) matched those previously reported (see Supporting Information File 1).

## Supporting Information

File 1 Starting materials and Copies of ^1^H and ^13^C NMR spectra of all products.
