# Supplementary material for: Selective carboxylation of reactive benzylic C–H bonds by a hypervalent iodine(III)/inorganic bromide oxidation system
Source: Beilstein J Org Chem. 2018 May 16;14:1087–94. doi: 10.3762/bjoc.14.94 (PMC6009330; doi:10.3762/bjoc.14.94)
Supplement: File 1 — Starting materials and Copies of 1H and 13C NMR spectra of all products. [file Beilstein_J_Org_Chem-14-1087-s001.pdf]

Supporting Information

for

Selective carboxylation of reactive benzylic C–H bonds by a hypervalent iodine(III)/inorganic bromide oxidation system

Toshifumi Dohi*[1], Shohei Ueda[1], Kosuke Iwasaki[1], Yusuke Tsunoda[1], Koji Morimoto[1], and Yasuyuki Kita*[2]

Address: [1]College of Pharmaceutical Sciences, Ritsumeikan University, 1-1-1 Nojihigashi, Kusatsu, Shiga 525-8577 Japan. Tel: +81-77-561-4908, and [2]Research Organization of Science and Technology, Ritsumeikan University, 1-1-1 Nojihigashi, Kusatsu, Shiga 525-8577 Japan. Tel & Fax: +81-77-561-5829

Email: Toshifumi Dohi* - td1203@ph.ritsumei.ac.jp; Yasuyuki Kita* - kita@ph.ritsumei.ac.jp

* Corresponding author

Starting materials and Copies of ^{1}H and ^{13}C NMR spectra of all products

Table of contents

Starting materials S2

Copies of ^{1}H and ^{13}C NMR spectra S3

References S16

Starting materials

1-Ethyl-4-methoxy-3-thiocyanatobenzene (**1f**) was prepared from 1-ethyl-4-methoxybenzene (**1a**) through hypervalent iodine(III)-induced aromatic cation radical coupling with thiocyanate according to literature [1,2]. Other chemicals (substrates, reagents, and anhydrous solvent, etc.) employed in this study were purchased from commercial suppliers and used as received without further purification.

Copies of ^{1}H and ^{13}C NMR spectra

^{1}H NMR (400 MHz, $CDCl_3$)

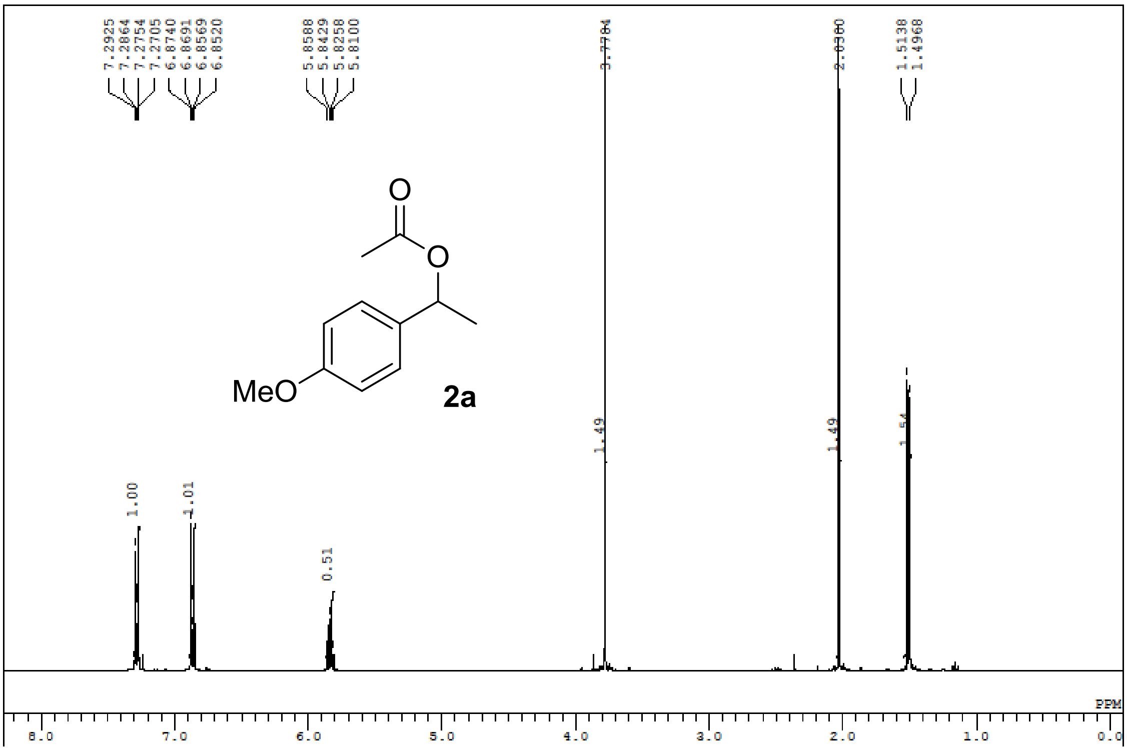

^{13}C NMR (100 MHz, $CDCl_3$)

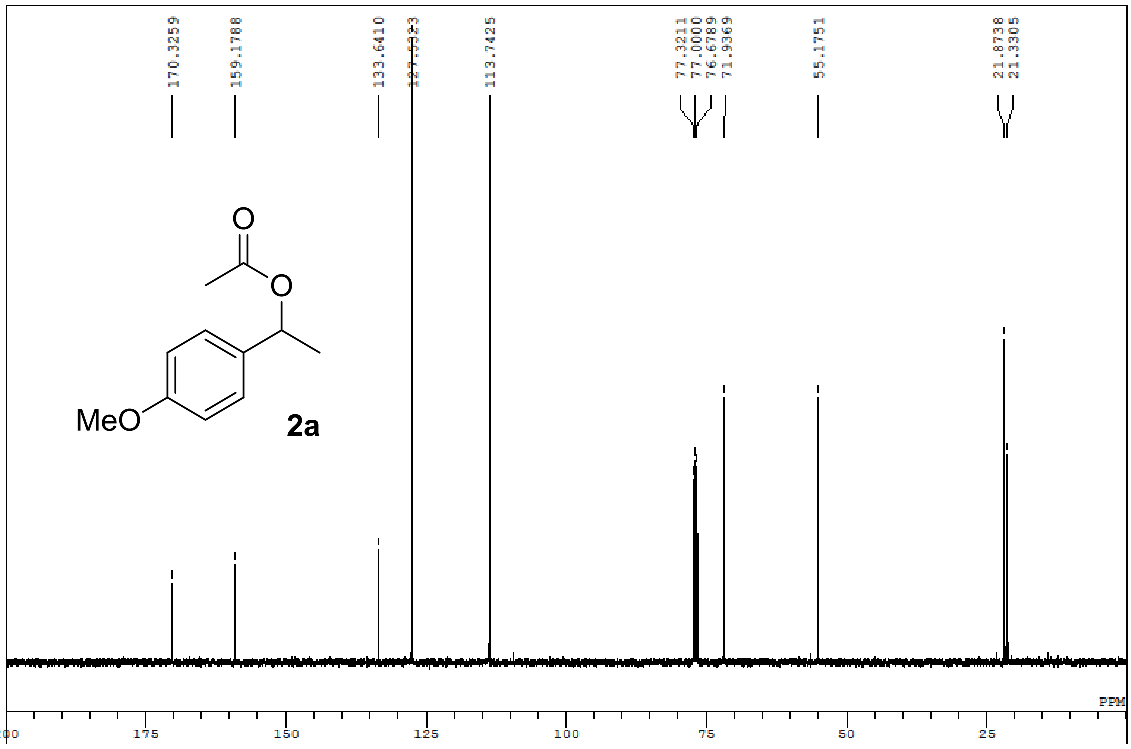

^{1}H NMR (400 MHz, $CDCl_3$)

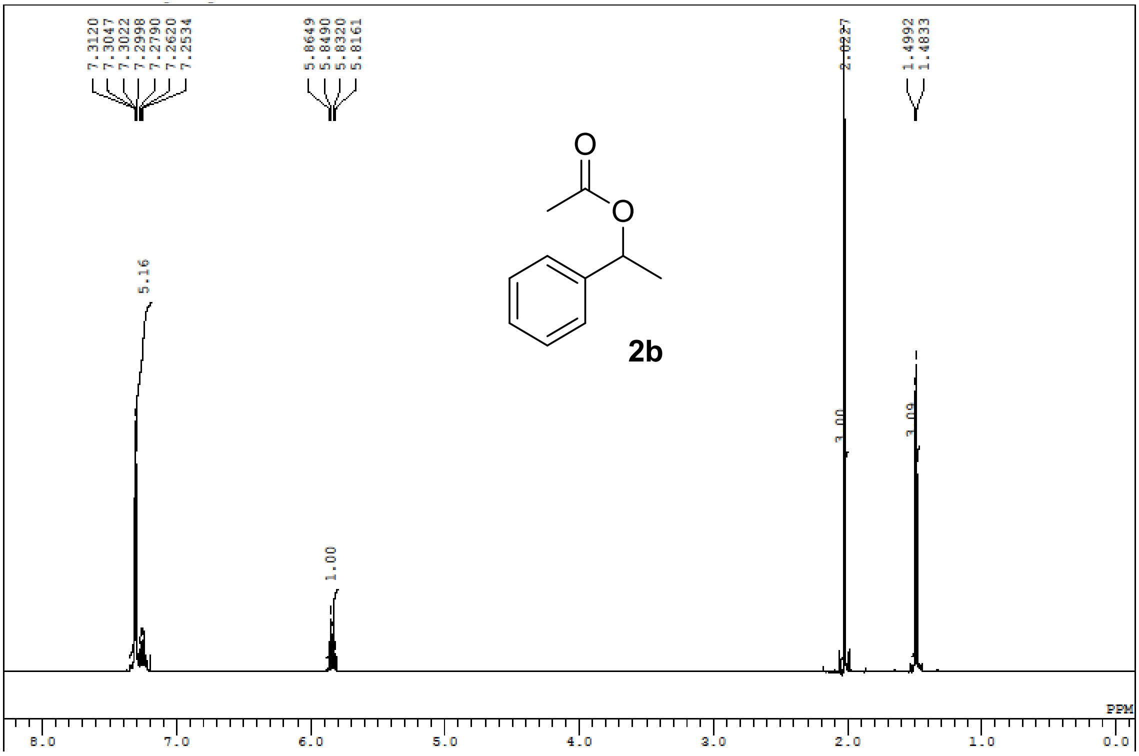

^{13}C NMR (100 MHz, $CDCl_3$)

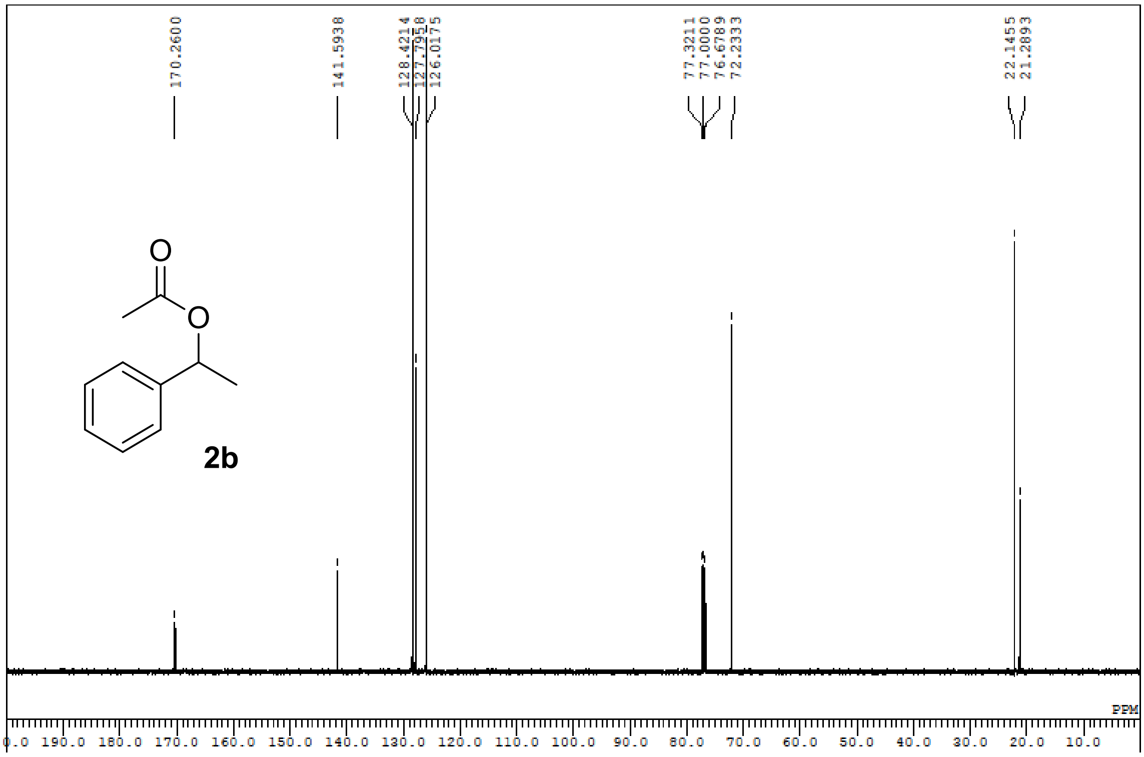

^{1}H NMR (400 MHz, $CDCl_3$)

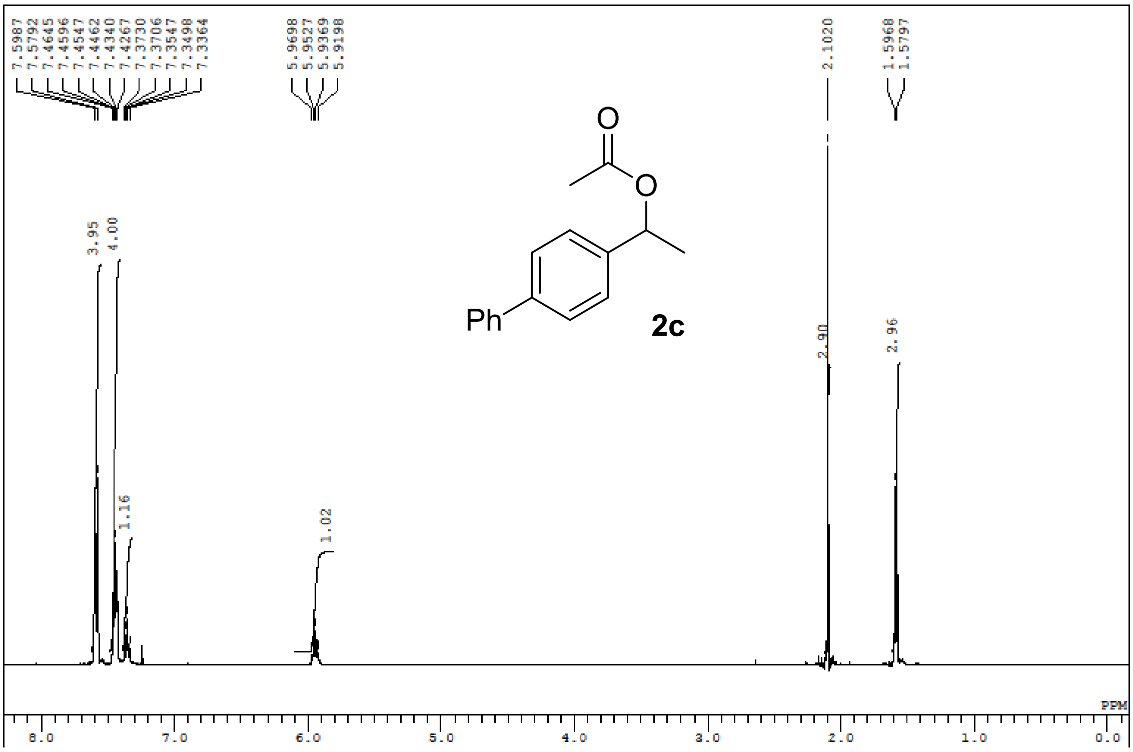

^{13}C NMR (100 MHz, $CDCl_3$)

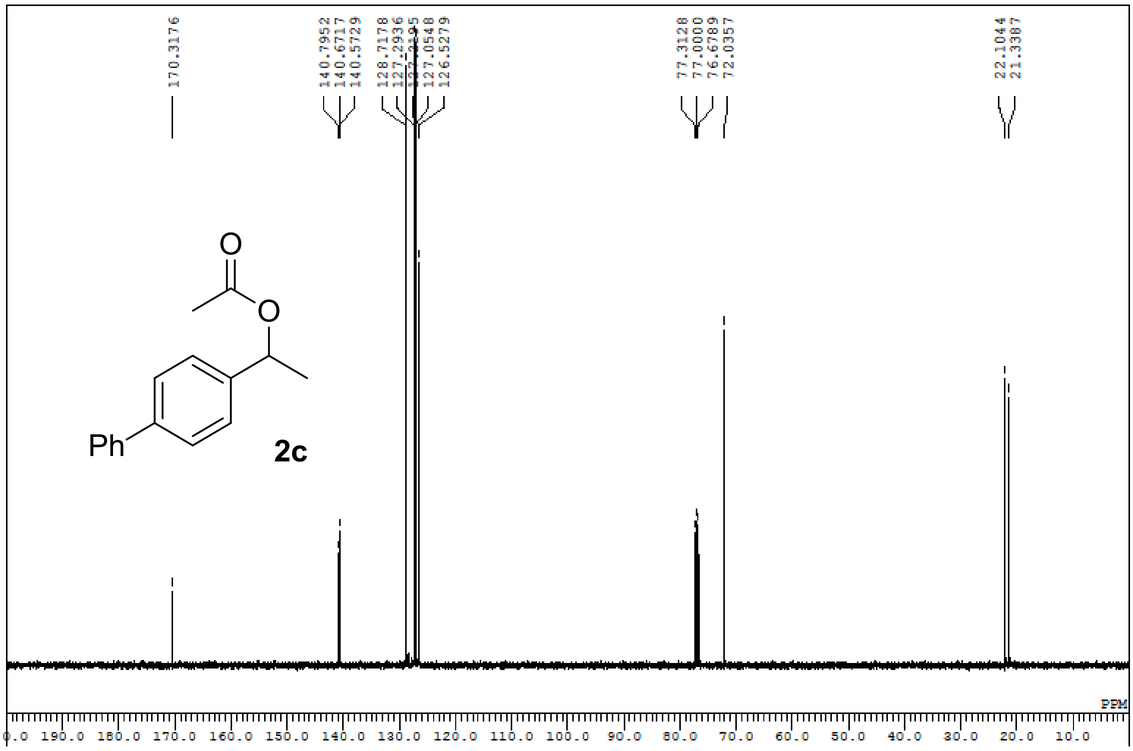

^{1}H NMR (400 MHz, $CDCl_3$)

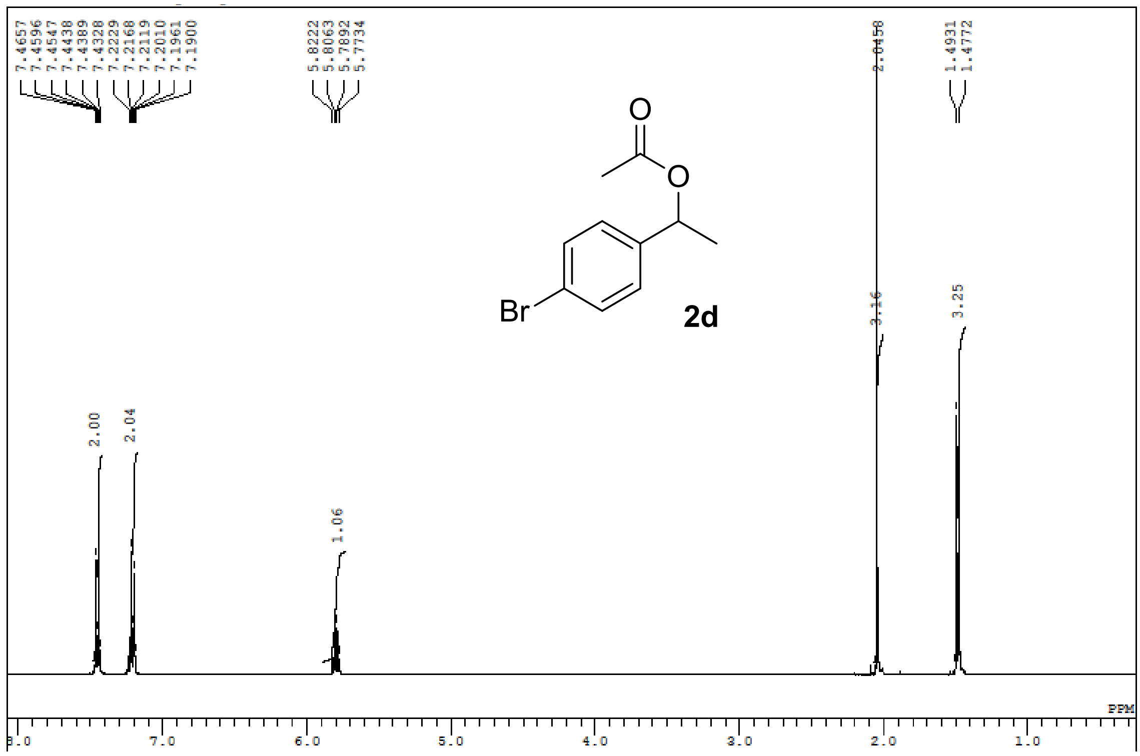

^{13}C NMR (100 MHz, $CDCl_3$)

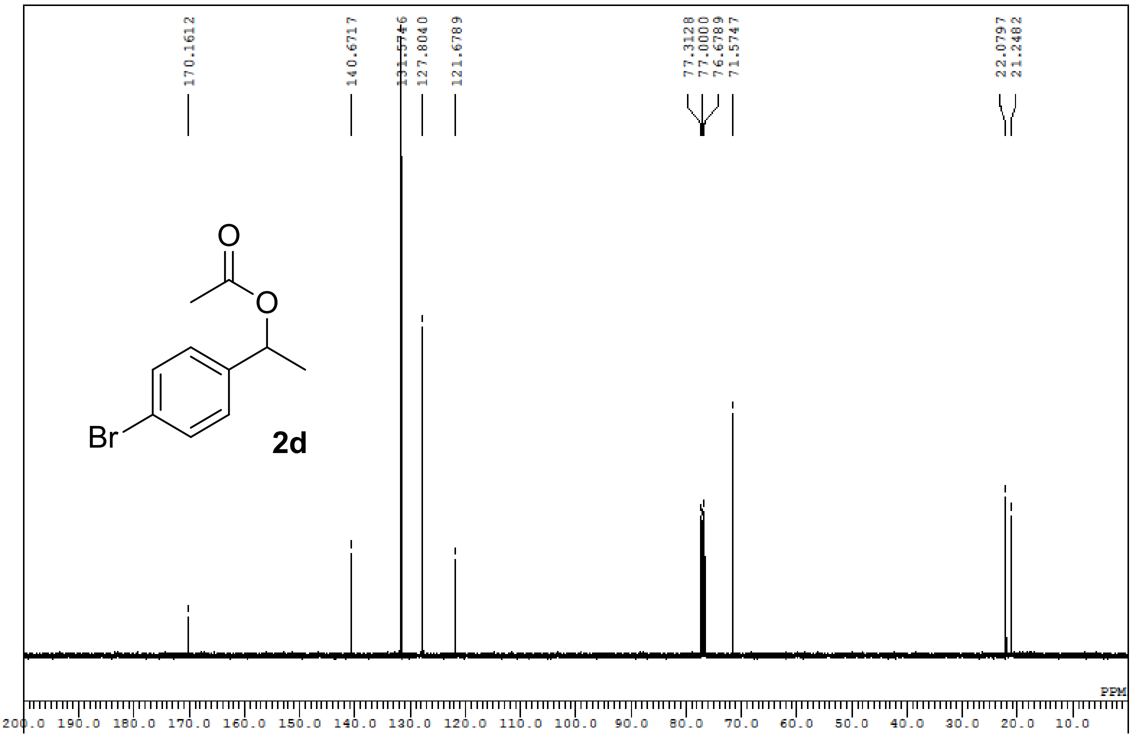

^{1}H NMR (400 MHz, $CDCl_3$)

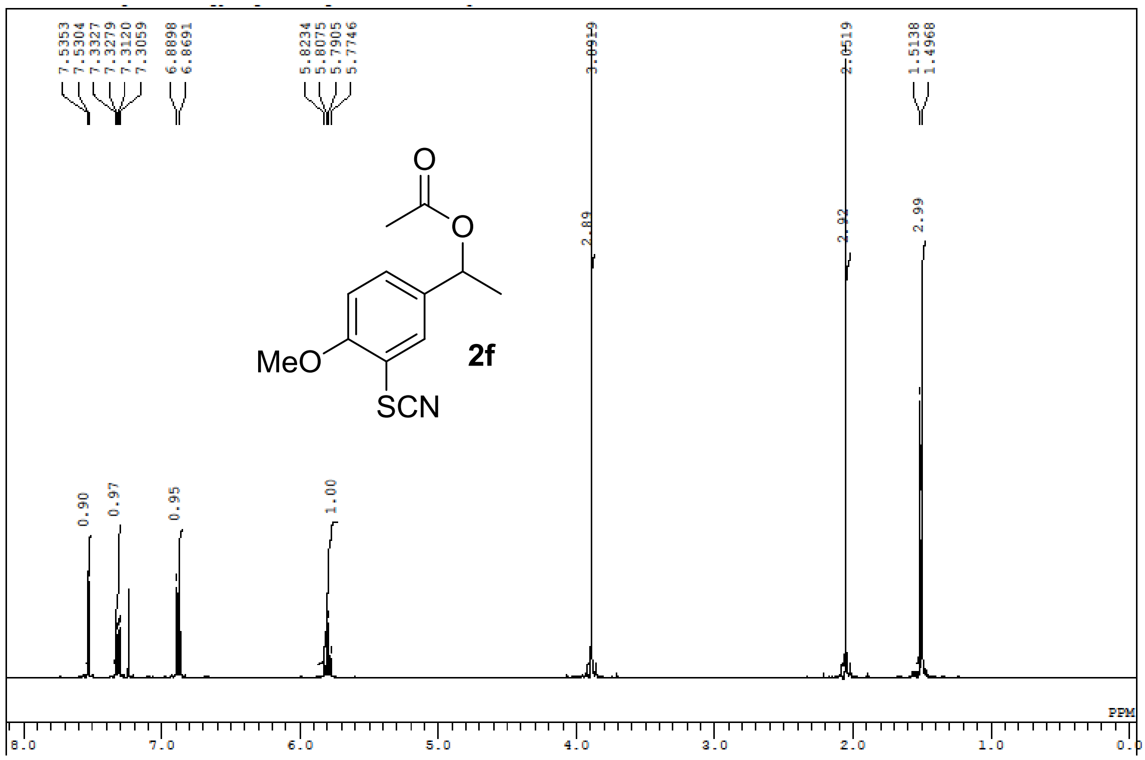

^{13}C NMR (100 MHz, $CDCl_3$)

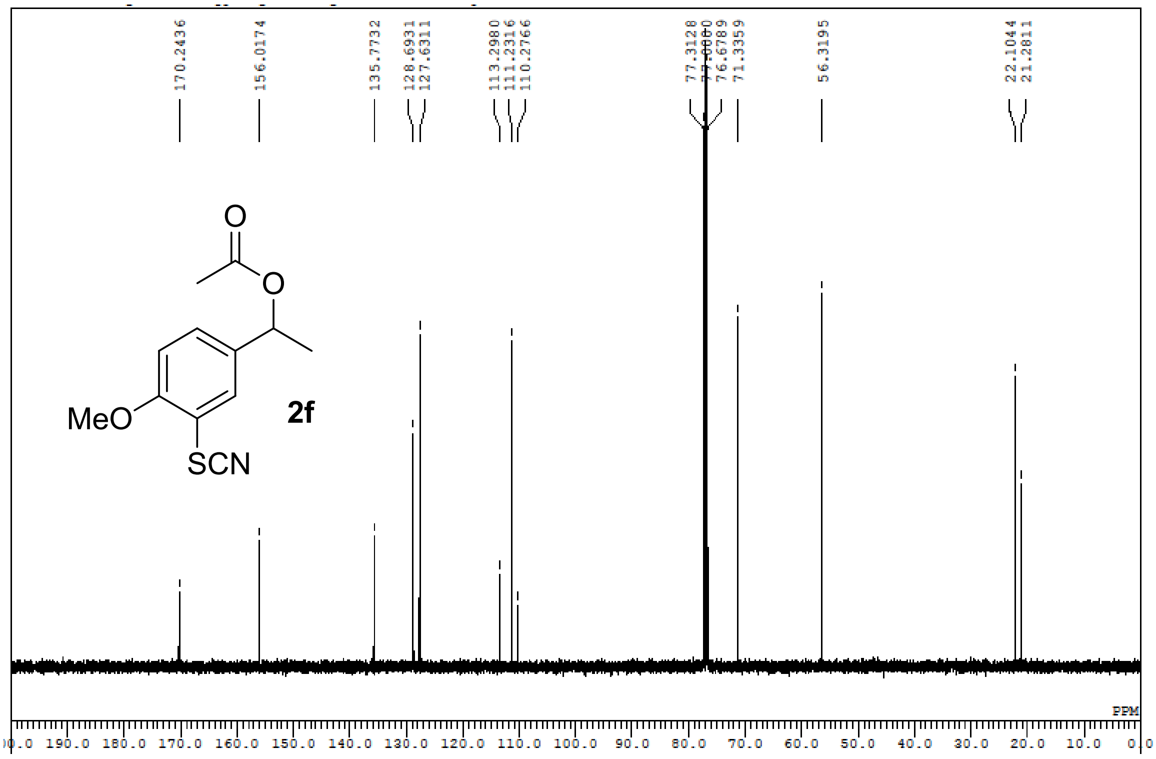

^{1}H NMR (400 MHz, $CDCl_3$)

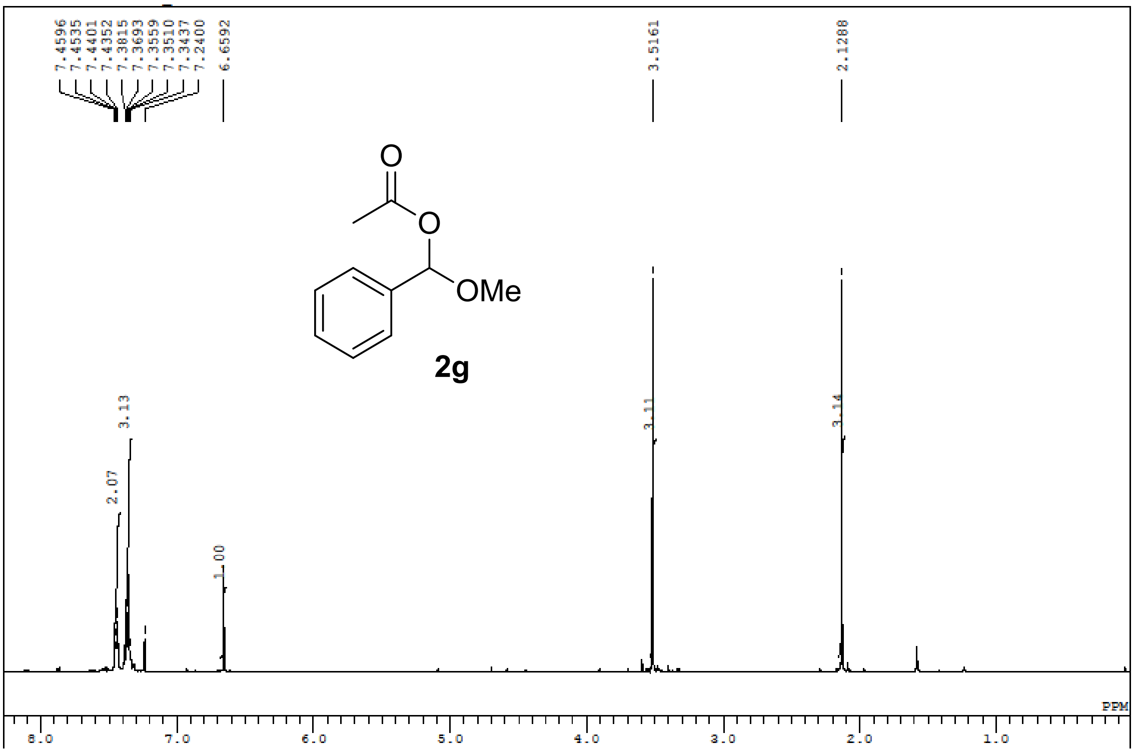

^{13}C NMR (100 MHz, $CDCl_3$)

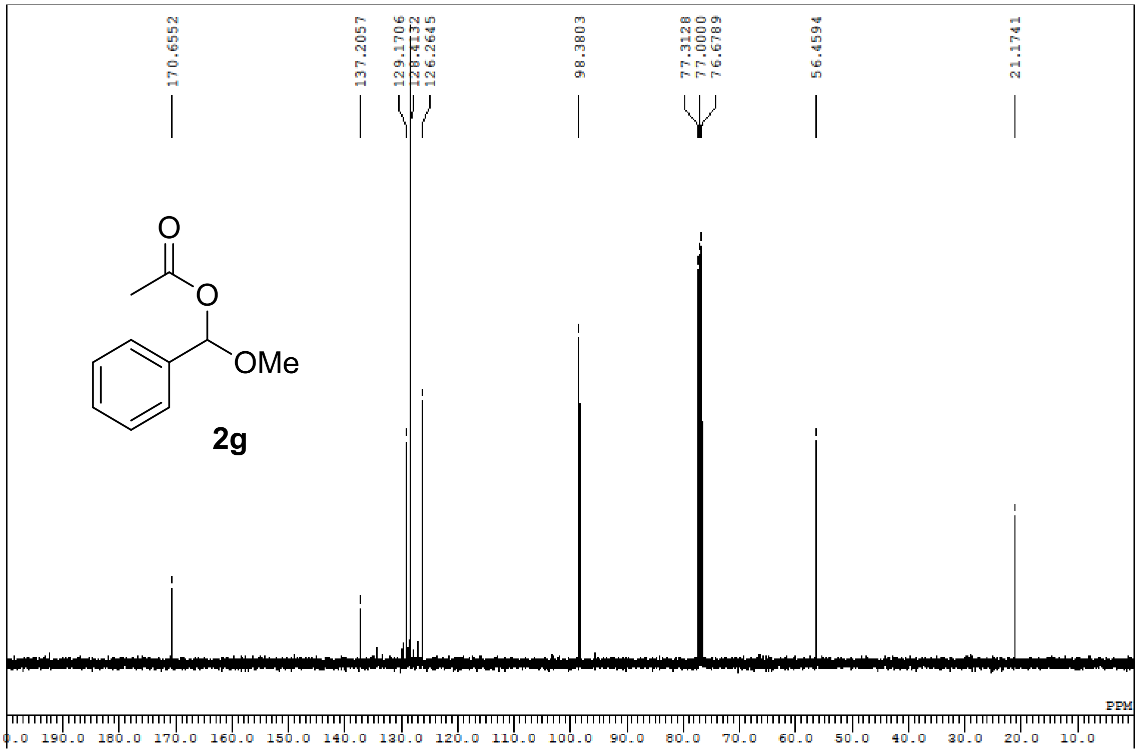

^{1}H NMR (400 MHz, $CDCl_3$)

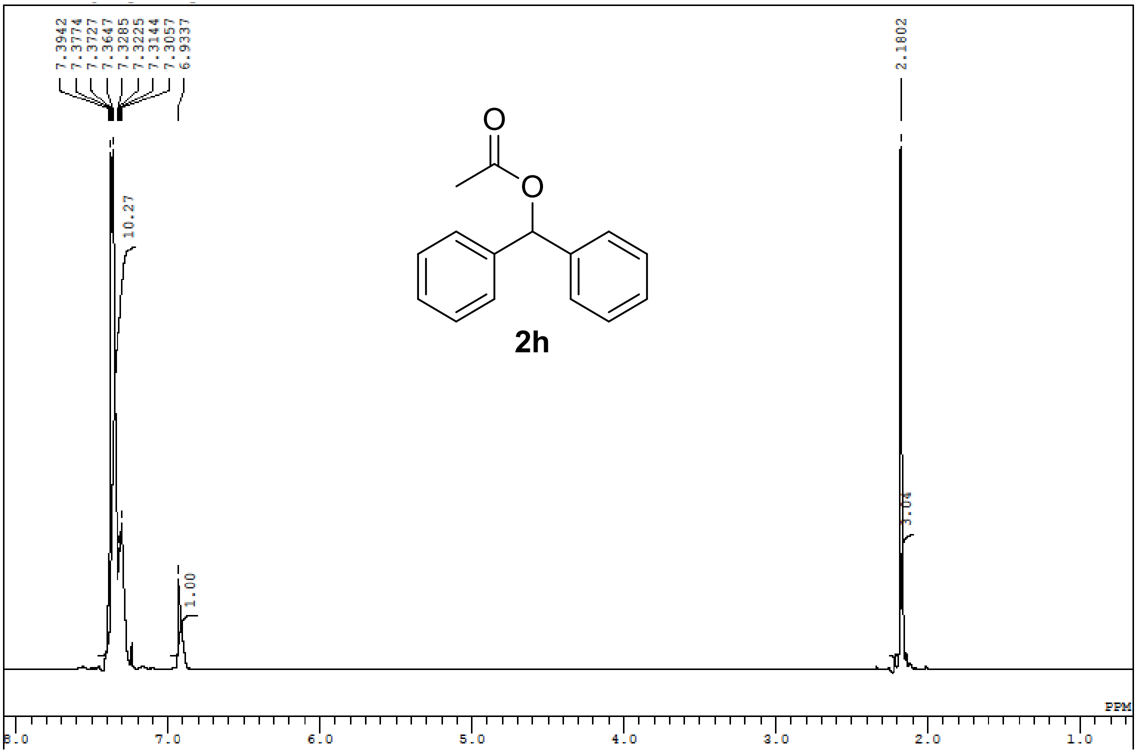

^{13}C NMR (100 MHz, $CDCl_3$)

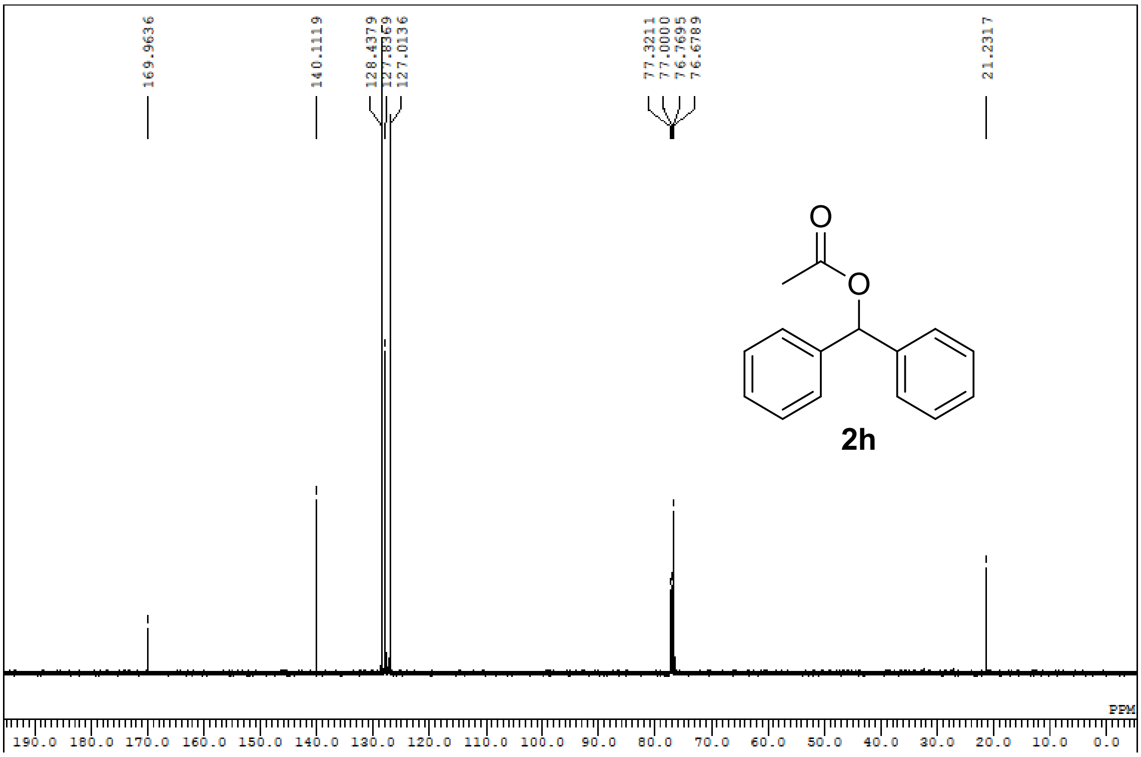

^1H NMR (400 MHz, $CDCl_3$)

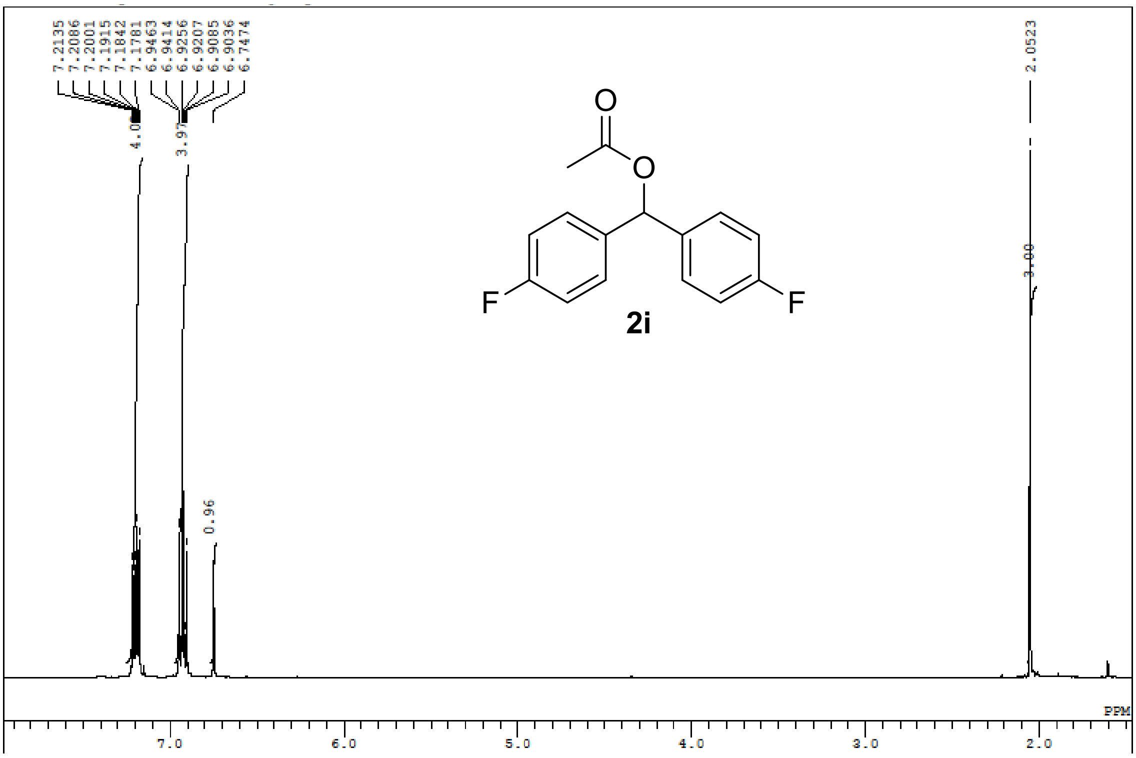

^{13}C NMR (100 MHz, $CDCl_3$)

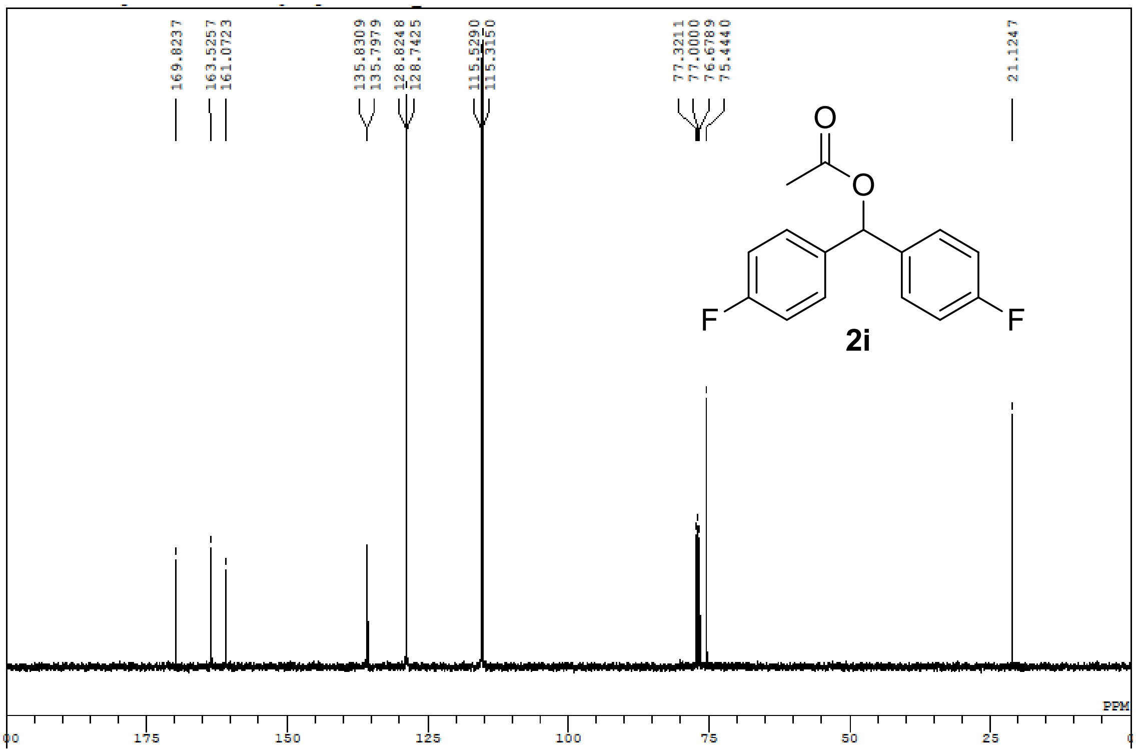

^{1}H NMR (400 MHz, $CDCl_3$)

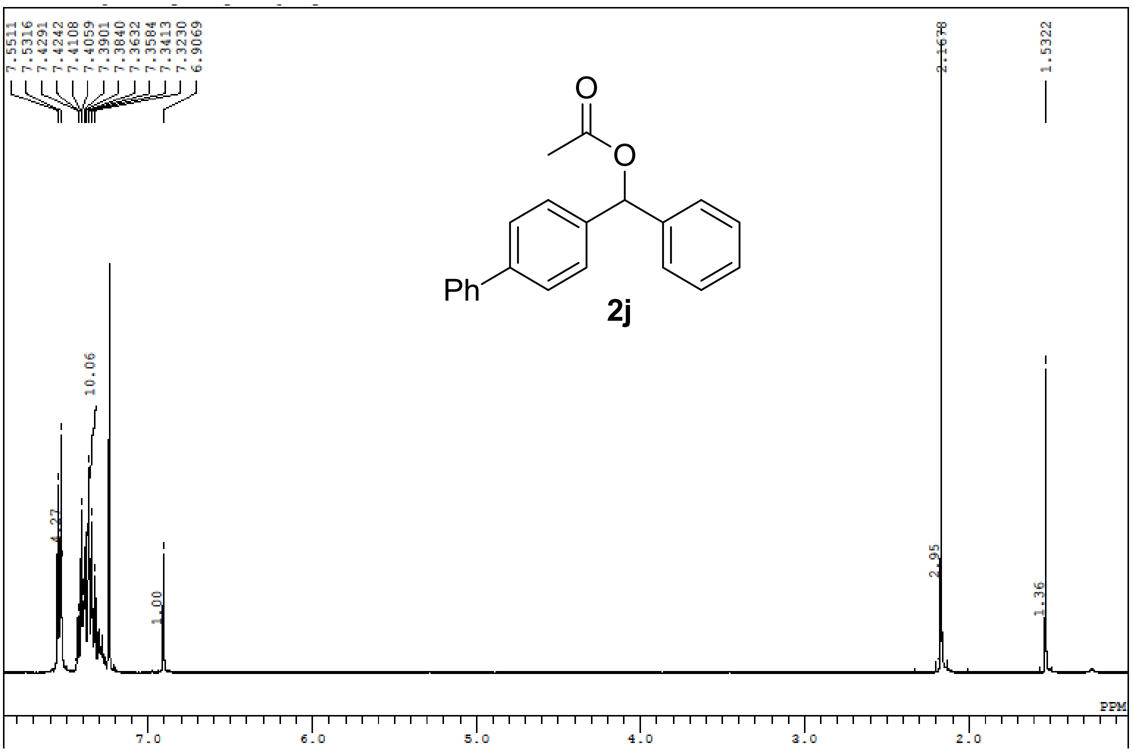

^{13}C NMR (100 MHz, $CDCl_3$)

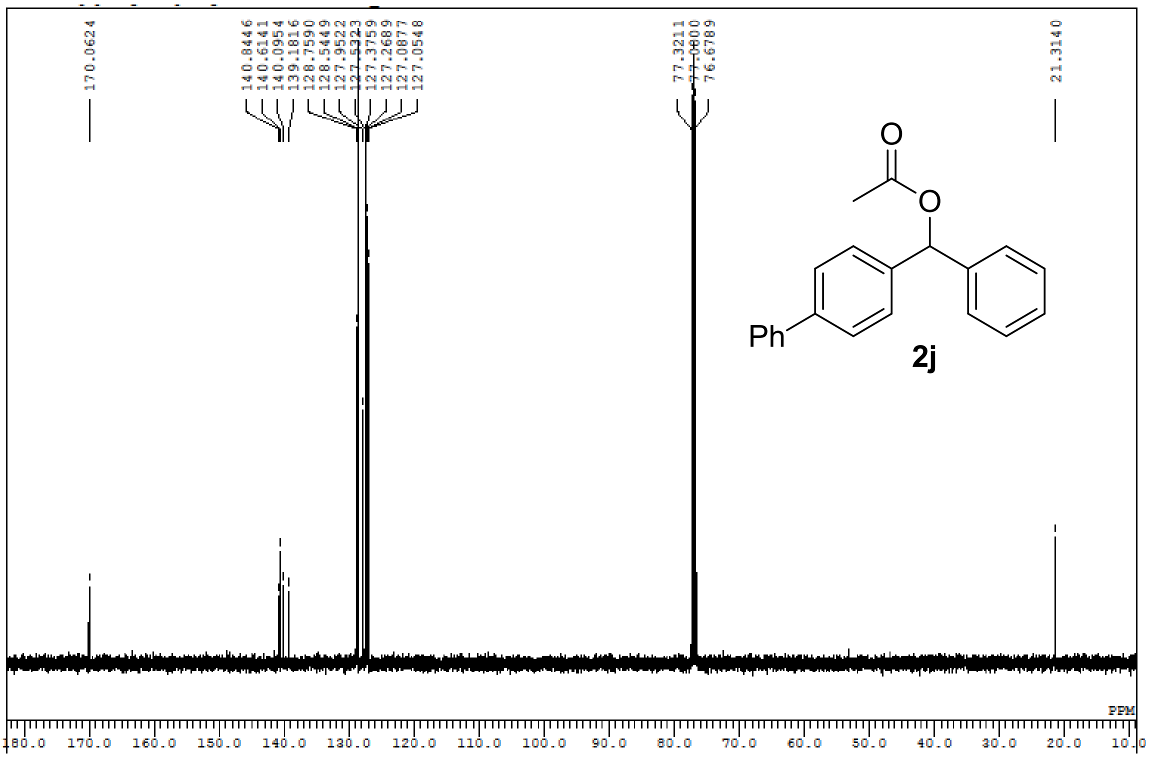

^{1}H NMR (400 MHz, $CDCl_3$)

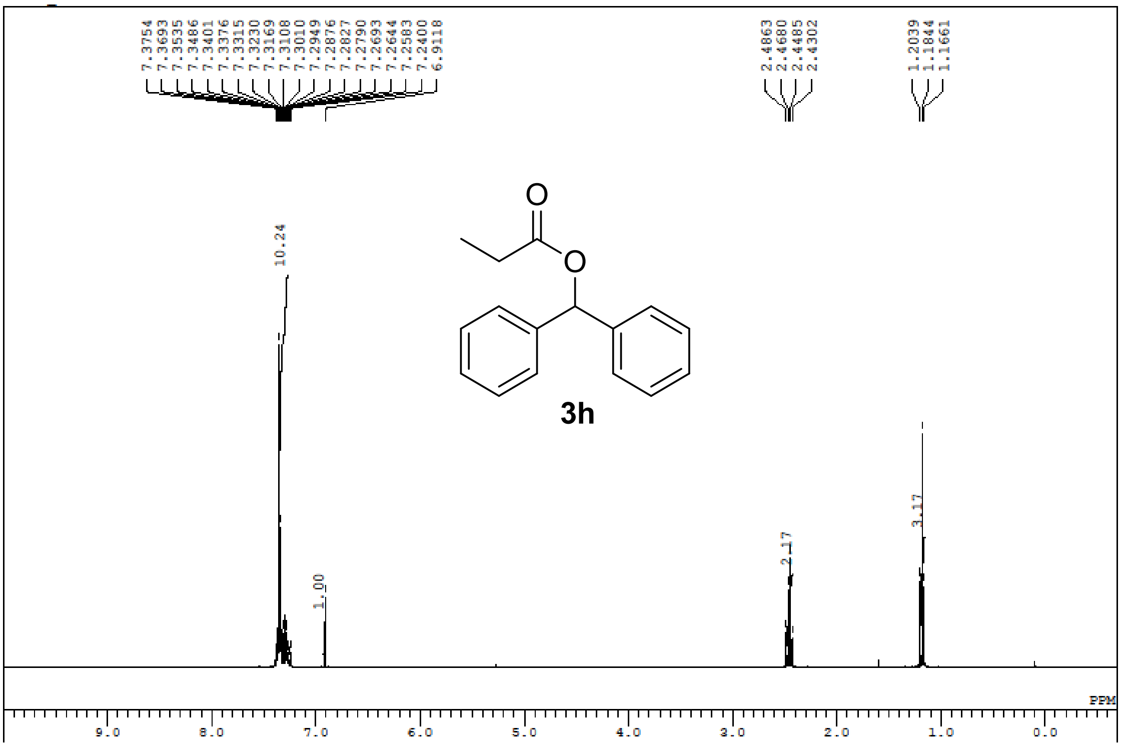

^{13}C NMR (100 MHz, $CDCl_3$)

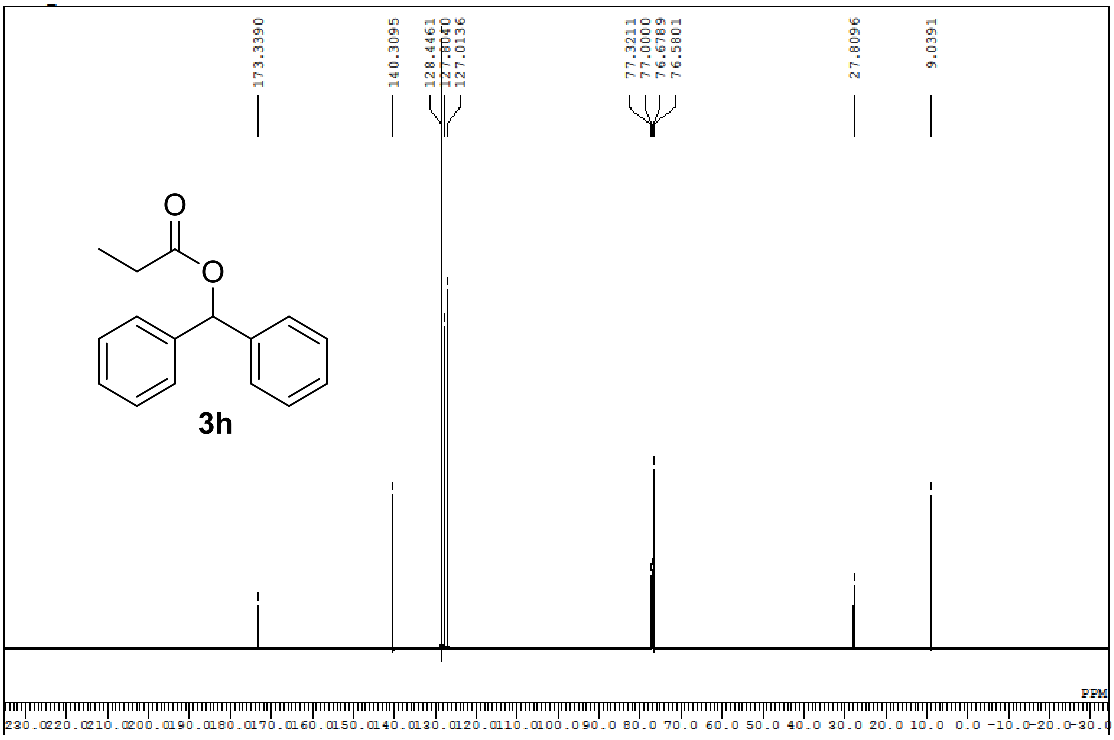

^{1}H NMR (400 MHz, $CDCl_3$)

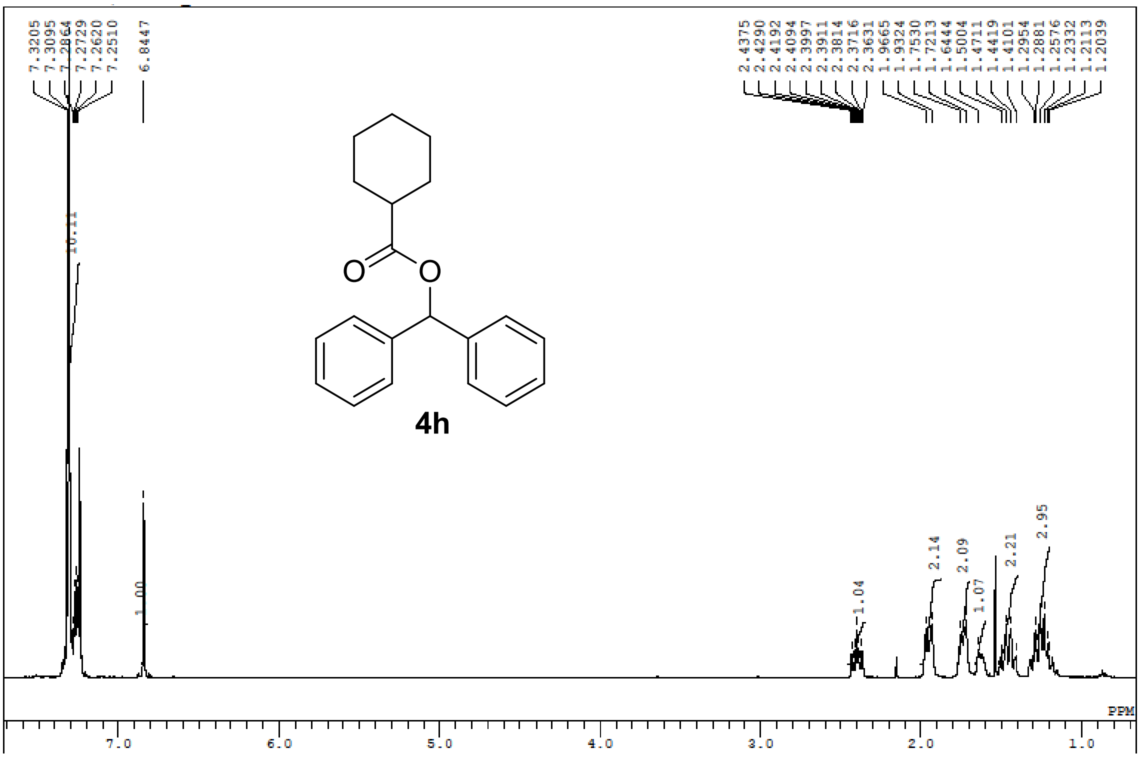

^{13}C NMR (100 MHz, $CDCl_3$)

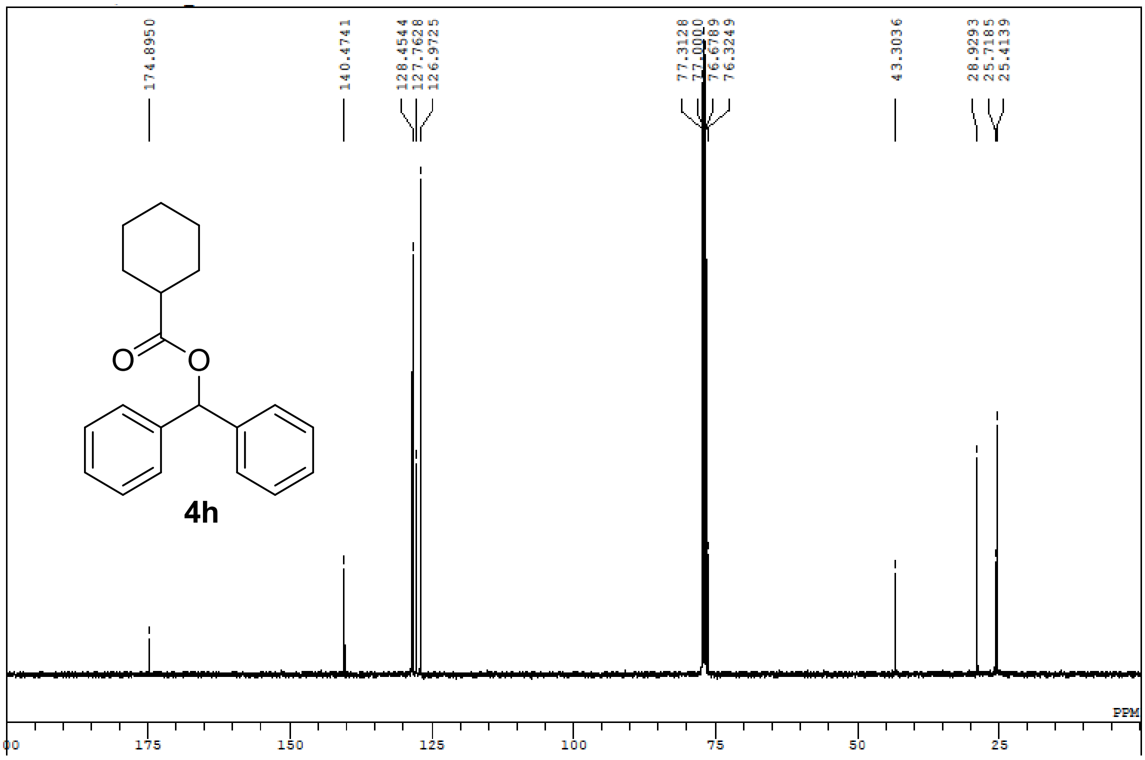

^{1}H NMR (400 MHz, $CDCl_3$)

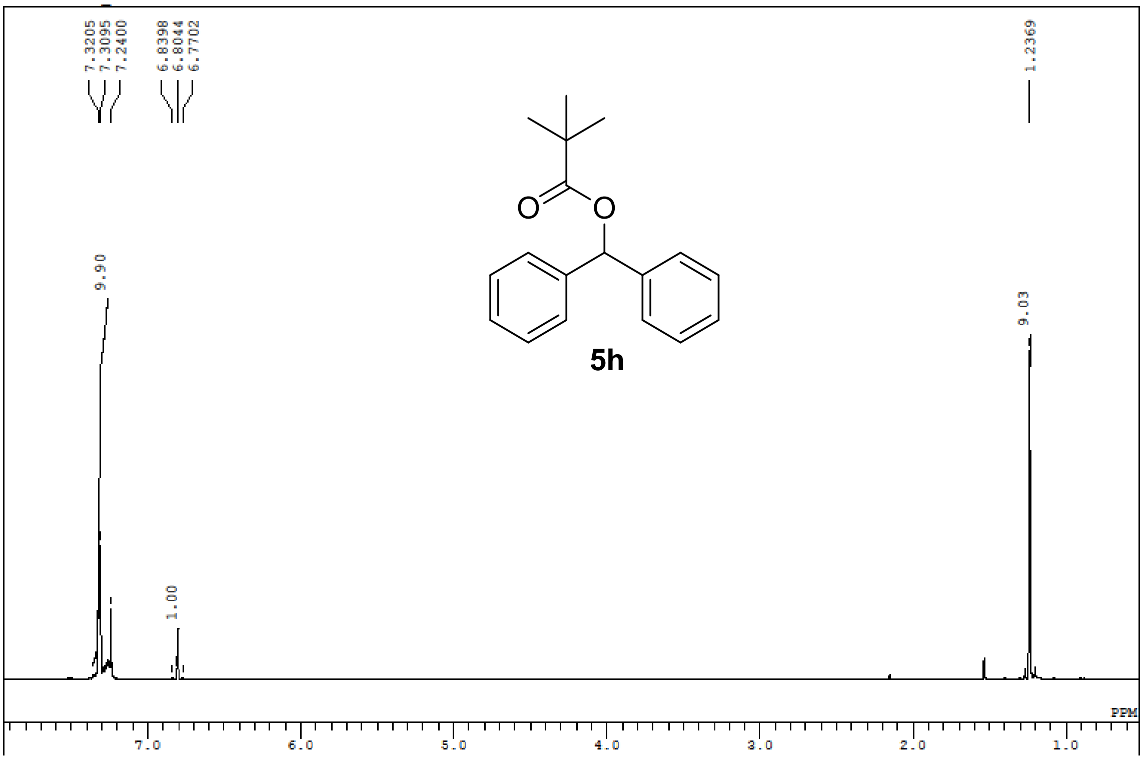

^{13}C NMR (100 MHz, $CDCl_3$)

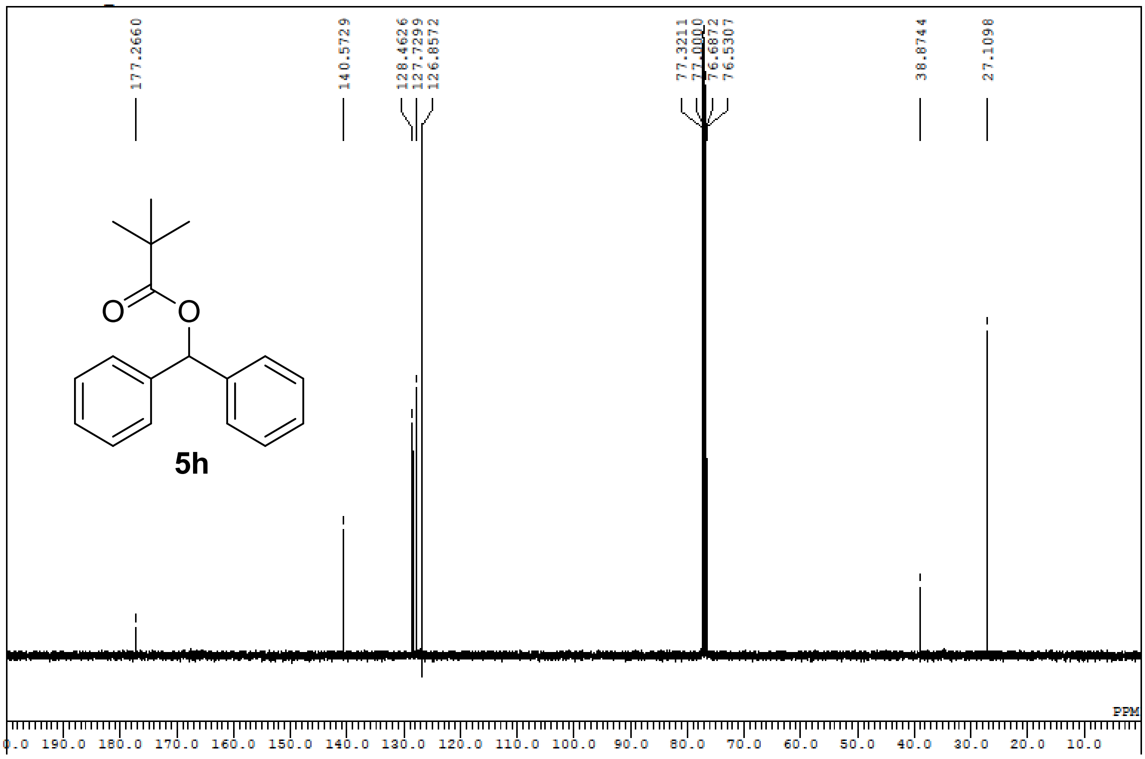

^{1}H NMR (400 MHz, $CDCl_3$)

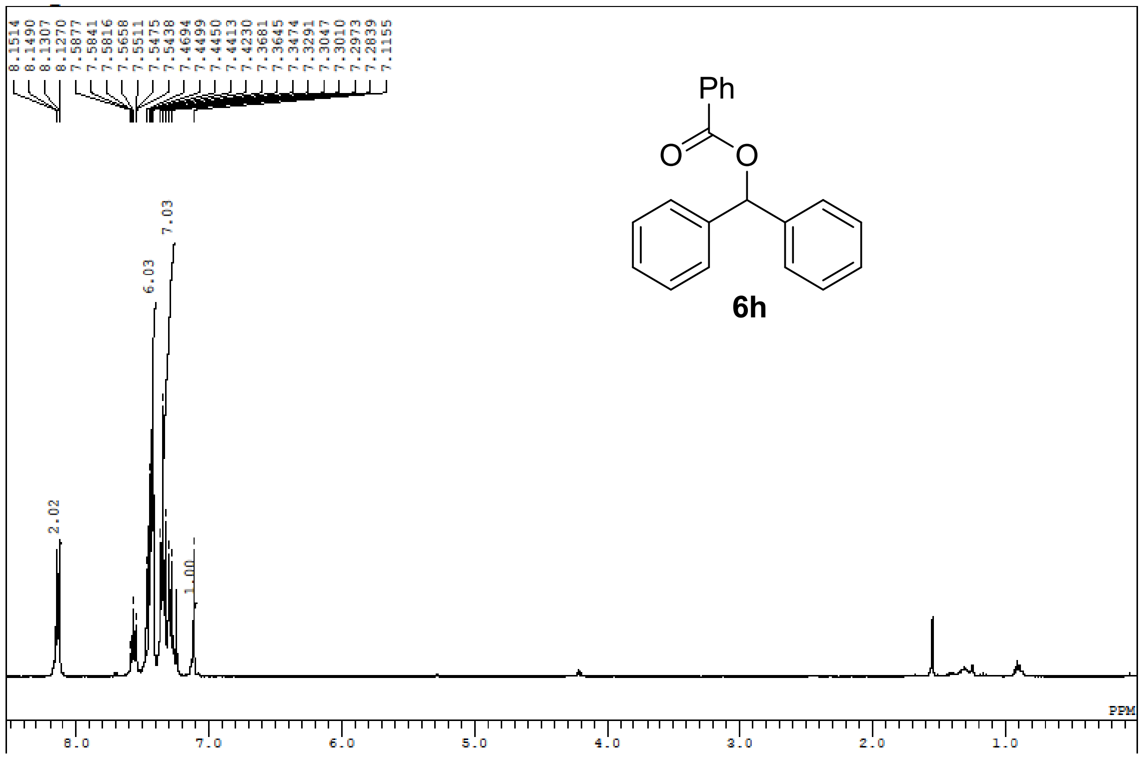

^{13}C NMR (100 MHz, $CDCl_3$)

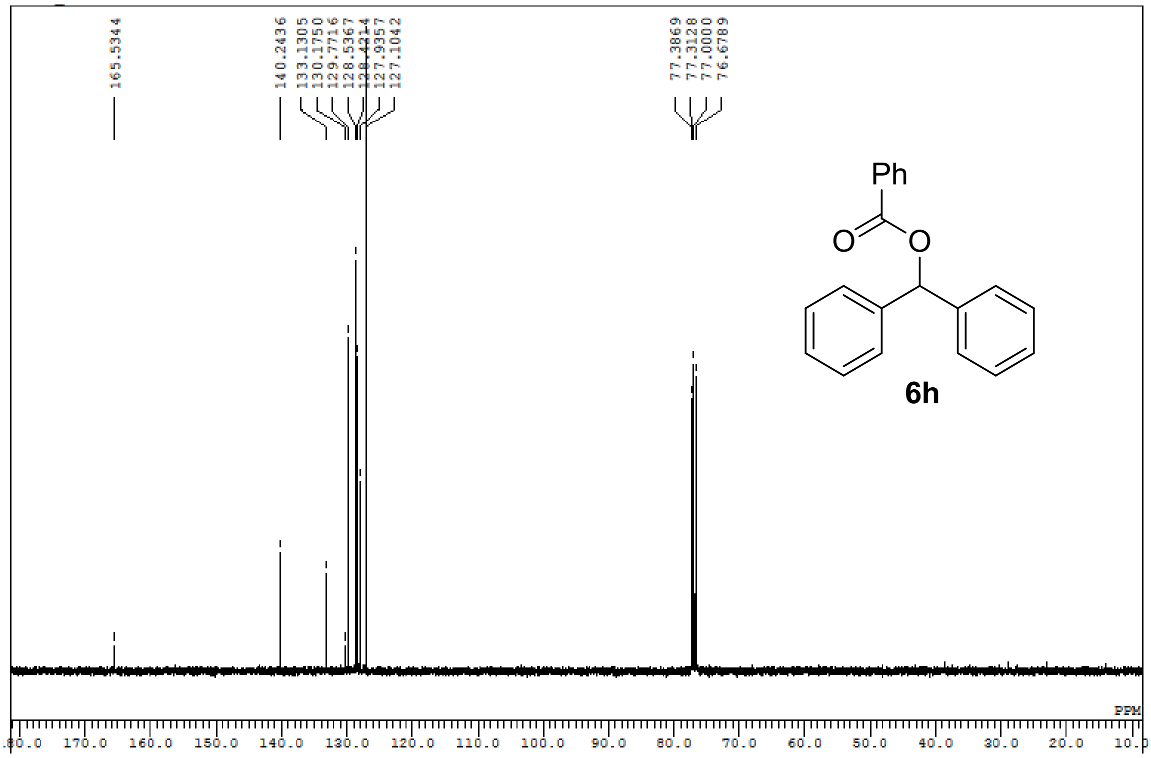

We confirmed that the physical and spectral data of the products (**2a–d, 2g–j, 3h, 4h, 5h, 6h**) matched those of the authentic samples (see the following references [3-13]).

References

1. Kita, Y.; Tohma, H.; Hatanaka, K.; Takada, T.; Fujita, S.; Mitoh, S.; Sakurai, H.; Oka, S. *J. Am. Chem. Soc.* **1994**, *116*, 3684.
2. Kita, Y.; Takada, T.; Mihara, S.; Whelan, B. A.; Tohma, H. *J. Org. Chem.* **1995**, *60*, 7144.
3. Product **2a**: Sokol, W.; Warkentin, J. *Can. J. Chem.* **2006**, *84*, 927.
4. Product **2b**: Magens, S.; Ertelt, M.; Jatsch, A.; Plietker, B. *Org. Lett.* **2008**, *10*, 53.
5. Product **2c**: Hatzakis, N. S.; Smonou, I. *Bioorg. Chem.* **2005**, *33*, 325.
6. Product **2d** and **6h**: Baba, H.; Moriyama, K.; Togo, H. *Tetrahedron Lett.* **2011**, *52*, 4303.
7. Product **2g**: Maziarz, E.; Furman, B. *Tetrahedron* **2014**, *70*, 1651.
8. Product **2h**: Magens, S.; Plietker, B. *J. Org. Chem.* **2010**, *75*, 3715.
9. Product **2i**: Laurent, M.; Marchand-Brynaert, J. *Synthesis* **2000**, 667.
10. Product **2j**: Engstroem, K.; Vallin, M.; Hult, K.; Baeckvall, J.-E. *Tetrahedron* **2012**, *68*, 7613.
11. Product **3h**: Zhang, L.; Luo, Y.; Fan, R.; Wu, J. *Green Chem.* **2007**, *9*, 1022.
12. Product **4h**: Mukaiyama, T.; Oohashi, Y.; Fukumoto, K. *Chem. Lett.* **2004**, *33*, 552.
13. Product **5h**: Squitieri, R. A.; Shearn-Nance, G. P.; Hein, J. E.; Shaw, J. T. *J. Org. Chem.* **2016**, *81*, 5278.
